# Climate warming and increasing *Vibrio vulnificus* infections in North America

**DOI:** 10.1038/s41598-023-28247-2

**Published:** 2023-03-23

**Authors:** Elizabeth J. Archer, Craig Baker-Austin, Timothy J. Osborn, Natalia R. Jones, Jaime Martínez-Urtaza, Joaquín Trinanes, James D. Oliver, Felipe J. Colón González, Iain R. Lake

**Affiliations:** 1 School of Environmental Sciences, University of East Anglia, Norwich, Norfolk UK; 2 Centre for Environment Fisheries and Aquaculture Science, Weymouth, Dorset UK; 3 Autonomous University of Barcelona, Barcelona, Spain; 4 University of North Carolina at Charlotte, Charlotte, USA; 5 CRETUS, Department Electronics and Computer Science, Universidade de Santiago de Compostela, Campus Universitario Sur, Santiago de Compostela, Spain; 6 National Oceanic and Atmospheric Administration, Atlantic Oceanographic and Meteorological Laboratory, 4301 Rickenbacker Causeway, Miami, FL 33149 USA; 7 Rosenstiel School of Marine and Atmospheric Science, University of Miami, Cooperative Institute for Marine and Atmospheric Studies, 4600 Rickenbacker Causeway, Miami, FL 33149 USA; 8 Data for Science and Health, Wellcome Trust, London, UK; 9 Department of Infectious Disease Epidemiology, Centre for Mathematical Modelling of Infectious Diseases, London School of Hygiene & Tropical Medicine, London, UK

**Keywords:** Climate change, Infectious diseases, Water microbiology

## Abstract

*Vibrio vulnificus* is an opportunistic bacterial pathogen, occurring in warm low-salinity waters. *V. vulnificus* wound infections due to seawater exposure are infrequent but mortality rates are high (~ 18%). Seawater bacterial concentrations are increasing but changing disease pattern assessments or climate change projections are rare. Here, using a 30-year database of *V. vulnificus* cases for the Eastern USA, changing disease distribution was assessed. An ecological niche model was developed, trained and validated to identify links to oceanographic and climate data. This model was used to predict future disease distribution using data simulated by seven Global Climate Models (GCMs) which belong to the newest Coupled Model Intercomparison Project (CMIP6). Risk was estimated by calculating the total population within 200 km of the disease distribution. Predictions were generated for different “pathways” of global socioeconomic development which incorporate projections of greenhouse gas emissions and demographic change. In Eastern USA between 1988 and 2018, *V. vulnificus* wound infections increased eightfold (10–80 cases p.a.) and the northern case limit shifted northwards 48 km p.a. By 2041–2060, *V. vulnificus* infections may expand their current range to encompass major population centres around New York (40.7°N). Combined with a growing and increasingly elderly population, annual case numbers may double. By 2081–2100 *V. vulnificus* infections may be present in every Eastern USA State under medium-to-high future emissions and warming. The projected expansion of *V. vulnificus* wound infections stresses the need for increased individual and public health awareness in these areas.

## Introduction

Greenhouse gas emissions from human activity are changing our climate^1^. The global mean temperature has risen 1.2 °C since the pre-industrial period^2^. Despite the aim of the Paris Climate Agreement to limit this increase in global average temperature to “well below two degrees”^3^, 1.5 °C of warming may occur by the early 2030s^1^.

Impacts may be especially acute on the world’s coastlines which provide a major interface between natural ecosystems and human populations and are a particular source of human disease. Vibrios are naturally occurring and commonly found Gram-negative bacteria in marine waters, which thrive in warm, brackish water and are highly sensitive to temperature^4^. These associations with climate have led to *Vibrio* species being collectively recognised as a “microbial barometer of climate change”^5^. Despite being endemic to subtropical regions (e.g. south-eastern USA^6^), *Vibrio* spp. infections have recently emerged at higher latitudes such as Delaware Bay, USA^6^ and the Baltic Sea^7^. The latter geographical shift has been formally attributed to climate change^8^. Recent modelling studies indicate that climate change will increase the suitability and distribution of pathogenic *Vibrio* species particularly at high latitudes^9^.

Of particular concern is *V. vulnificus* infection which can occur from exposure to seawater through small skin lesions^10^ and can quickly turn necrotic, requiring urgent surgical tissue removal or limb amputation in around 10% of cases^11^. *V. vulnificus* is the most pathogenic of the *Vibrio* genus: wound infection mortality rates are as high as 18%^12^ and fatalities have occurred as soon as 48 h following exposure^10^. Alongside causing around 100 cases annually in the USA^13^, the economic burden of *V. vulnificus* wound infections is estimated at over US$ 28 million/year^12^. Overall annual costs associated with this pathogen are estimated at US$ 320 million, making it the most expensive marine pathogen in the USA to treat^14^.

Despite a changing *V. vulnificus* infection distribution, there are few attempts to quantify this change, or to map the likely climate change effects. This is due to the lack of high-quality epidemiological data. Some studies have made future *Vibrio* spp. risk predictions based on the projected distribution of ideal environmental conditions (e.g.,^9^) but these predictions indicate the probable presence of *Vibrio* spp. bacteria rather than disease risk^13^.

Here, we produce a systematic assessment of the changing distribution of *V. vulnificus* infections along the east USA coast using a unique 30-year *V. vulnificus* infection database. The study area includes the USA Gulf Coast, a global hotspot for *V. vulnificus* infection^15^, and the Atlantic coastline where reported *V. vulnificus* infections are increasingly common^6^. We then explore the influence of climate change upon the spatial distribution of *V. vulnificus* wound infections using an Ecological Niche Model (ENM), a common tool for predicting species distribution based upon biogeographic variables and increasingly used for modelling disease transmission^16^.

Future changes to the *V. vulnificus* wound infection distribution were predicted using the ENM and future data simulated by seven Global Climate Models (GCMs) which belong to the newest Coupled Model Intercomparison Project (CMIP6)^17^. These GCM projections were available for different Shared Socioeconomic Pathways (SSPs)^17^ which enabled the influence of climate change in the *V. vulnificus* distribution to be assessed. SSPs offer an update to the previous Representative Concentration Pathways^18^ as they combine different socioeconomic “narratives”^19^, which shape trends including economic growth, population change and urbanisation with corresponding levels of greenhouse gas emissions by the end of the twenty-first century. We focus on scenarios SSP1-2.6 which is set against the SSP1 narrative of “sustainability” and is a low emissions scenario, and SSP3-7.0, which is set against the SSP3 socioeconomic backdrop of “regional rivalry”^20^ where resurgent nationalism and regional conflicts shift focus away from climate mitigation leading to medium-to-high emissions. These are referred to hereafter as SSP126 and SSP370. Analysis was based upon climate data (air temperature and precipitation) obtained from seven GCMs and oceanographic data (sea surface temperature and salinity) for one GCM (see “Methods” and Table S1 for GCM references).

## Results

### Changing incidence and distribution of *V. vulnificus* infections

**Figure 1:**
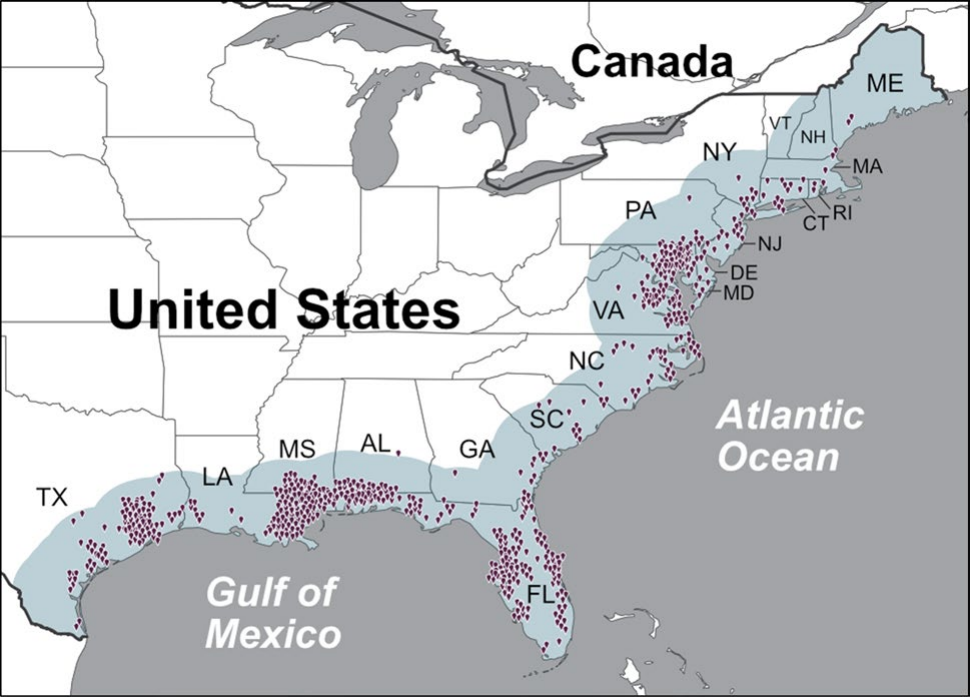
Original locations of the 709 confirmed non-foodborne *V. vulnificus* infections reported to the Cholera and Other *Vibrio* Illness Surveillance (COVIS) database between 2007 and 2018 within 200 km of the east USA coastline (blue shading).

**Figure 2:**
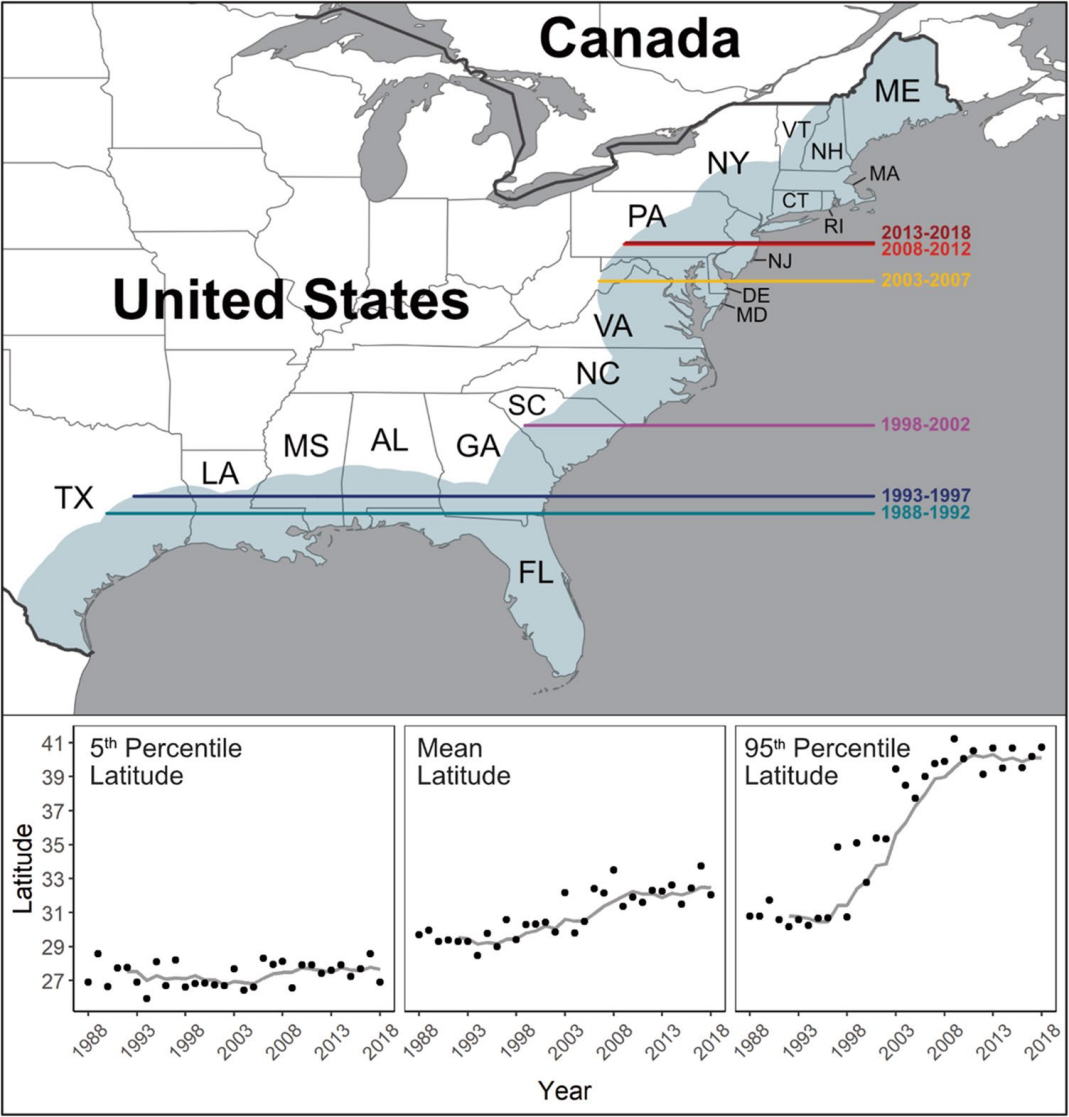
Latitudinal shifts by year of confirmed non-foodborne *V. vulnificus* infections in the USA reported to the Cholera and Other *Vibrio* Illness Surveillance (COVIS) between 1988 and 2018. The 95th latitude percentile in 5-year bands is presented in the upper panel. The lower panel presents the 5th percentile, mean and 95th latitude percentile for individual years alongside 5-year rolling means.

The historical distribution of *V. vulnificus* infections between 2007 and 2018 is presented in Fig. 1. This presents all cases where either the home or travel location was reported within 200 km of the eastern USA coastline. Cases were reported from the Mexican border along the entire coast of the USA to Maine. The total reported *V. vulnificus* cases has increased, from around 10 p.a. in 1988 to around 80 p.a. by 2018. Figure 2 presents the latitudinal distribution of *V. vulnificus* cases by year and shows that the 5th percentile of latitude (henceforth southern extent) of cases has remained constant at the Mexican border and not shifted northwards (linear trend p = 0.237), whereas the mean case latitude has moved northward at 0.13° (~ 15 km) p.a. (linear trend p < 0.001). The 95th latitude percentile (henceforth northern extent) of *V. vulnificus* cases has extended northwards at 0.43° (~ 48 km) p.a. (linear trend p < 0.001). On Fig. 2 the non-linear progression of the northern extent is likely a consequence of cases of this low probability disease only occurring when reaching high population density areas (e.g., Virginia, Maryland see Fig. 1).

Binary logistic regression models of the *V. vulnificus* infection distribution (presence-absence between the Mexican border and northern extent) were fitted using tenfold cross validation on 100 data subsets using generalised linear models (see “Methods”). Both mean air temperature (tmean) and maximum air temperature (tmax) were individually statistically significant (p < 0.001) with a high area under the receiver operator characteristic curve (AUC; 0.999 and 0.998, respectively) indicating good predictive accuracy for *V. vulnificus* infection presence. Mean precipitation and maximum precipitation were also significant (p < 0.001) but each had a lower AUC (0.833 and 0.687). Sea surface temperature and salinity were considered but the coarse dataset resolution (25 by 25 km vs 5 by 5 km for air temperature and precipitation) meant that they were unable to capture conditions close to the coast (where there can be sharp gradients particularly in salinity near to estuaries) especially as the effective resolution (i.e., including density of the underlying salinity observations) may be coarser. For example for every coastal grid cell (25 × 25 km) in the study area for every month, salinity values were never in the suitable range for *V. vulnificus* (2–25 Practical Salinity Units (PSU)^21^) (Supplementary Fig. S1), yet *V. vulnificus* infections are present in all these states (Fig. 1). This indicates that the true salinity of these near-shore waters is within the ideal range for *V. vulnificus*, but we are unable to observe the true salinity values due the coarse resolution of these data. This resulted in poor statistical relationships with *V. vulnificus*. Model fit was not improved by multivariable models hence the two models with the highest AUC were selected (i.e., tmean and tmax), to represent different temperature variations. These models were used to predict the *V. vulnificus* infection distribution into the future, using air temperature projections from seven GCMs.

### Future distribution and incidence of *V. vulnificus* infections

Projections are presented as maps of infection distribution, length of coastline within this distribution, population at risk within 200 km of the distribution and estimated annual case numbers. Results were produced for multiple time periods (2021–2040, 2041–2060, 2061–2080, 2081–2100) and SSPs (SSP126, SSP245, SSP370, SSP585). We focus on SSP126 and SSP370 to represent a range of likely socioeconomic trends and greenhouse gas emissions^22^. We focus on 2041–2060 and 2081–2100 to make our projection periods comparable with other impact modelling groups (e.g., The Inter-Sectoral Impact Model Intercomparison Project [ISIMIP]^23^). Results for other SSPs and time periods are found in Supplementary Information (Tables S2, S3 and Figs. S2–S9).

**Figure 3:**
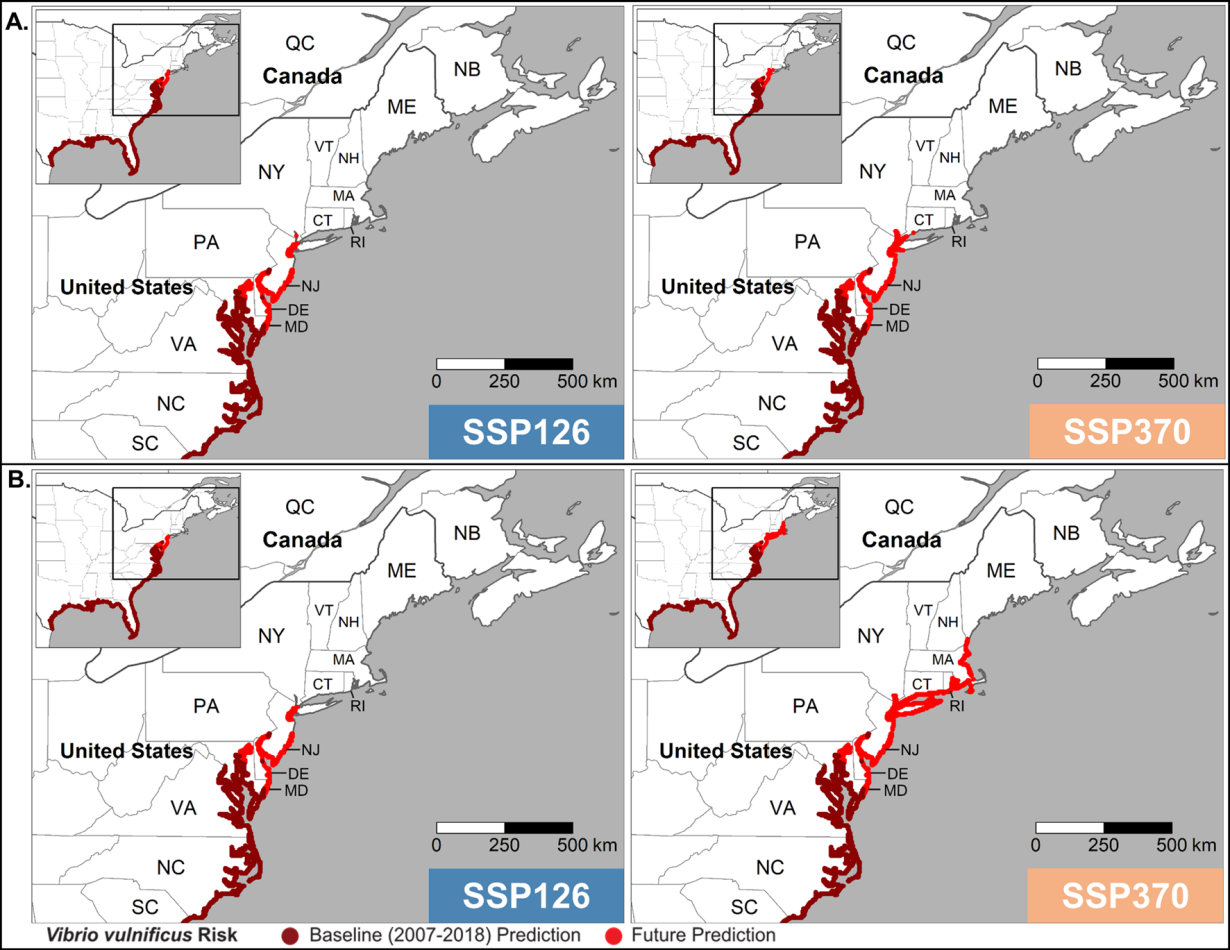
Baseline and projected *V. vulnificus* infection distribution (presence) predicted using tmean and then averaged across seven CMIP6 global climate models for (**A**) 2041–2060 and (**B**) 2081–2100 under CMIP6 SSP126 and SSP370.

**Figure 4:**
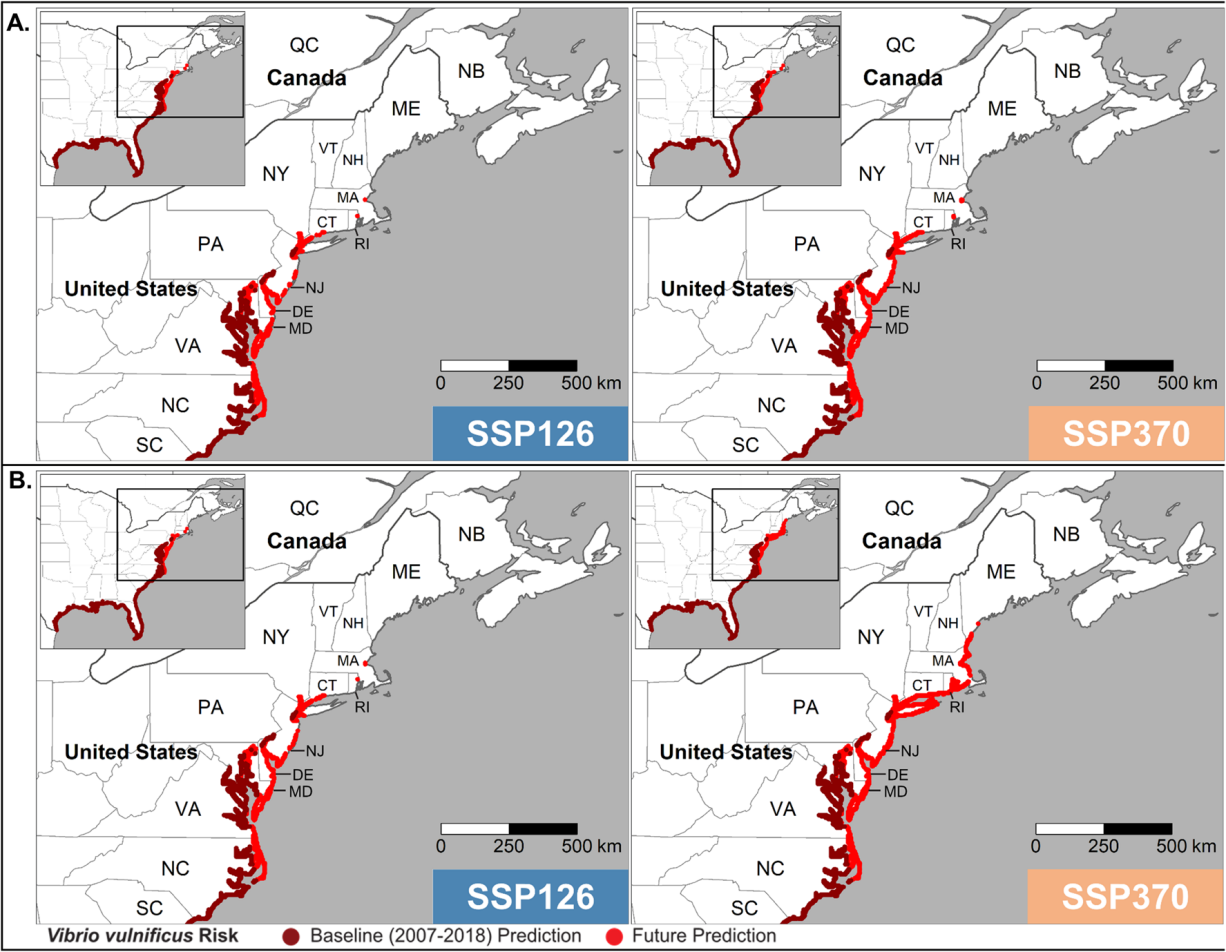
As Fig. 3 but predicted using tmax.

Tmean and tmax were the strongest predictors of infection distribution, and projections of future distribution from both are compared in Figs. 3 and 4. Projections were produced for both tmean and tmax to evaluate how sensitive the projections are to predictor choice. Baseline predictions are almost identical to the current distribution (Fig. 2), with the upper limit around Philadelphia (40.0°N). Under both SSP126 and SSP370, the tmean model (Fig. 3) predicts a northward expansion of *V. vulnificus* infection distribution to New Jersey (40.0°N) and southern New York state (40.9°N) by 2041–2060. Under SSP126, the projected distribution changes little from 2041–2060 to 2081–2100. Under SSP370 the distribution extends northwards to include the Connecticut (41.3°N), New Hampshire (43.0°N) and southern Maine (43.3°N) coastlines.

Under the tmax model (Fig. 4) by 2041–2060 SSP126 and SSP370 the *V. vulnificus* infection distribution extends further northward into Connecticut (41.3°N) and as far north as Boston, Massachusetts (42.4°N). By 2081–2100, there is a notable between-scenario difference with little further change in the distribution under SSP126 but an expansion towards the southern Maine coastline (43.3°N) under SSP370. Under SSP370, the distribution of *V. vulnificus* infections encompasses every Eastern USA coastal state by 2081–2100.

**Figure 5:**
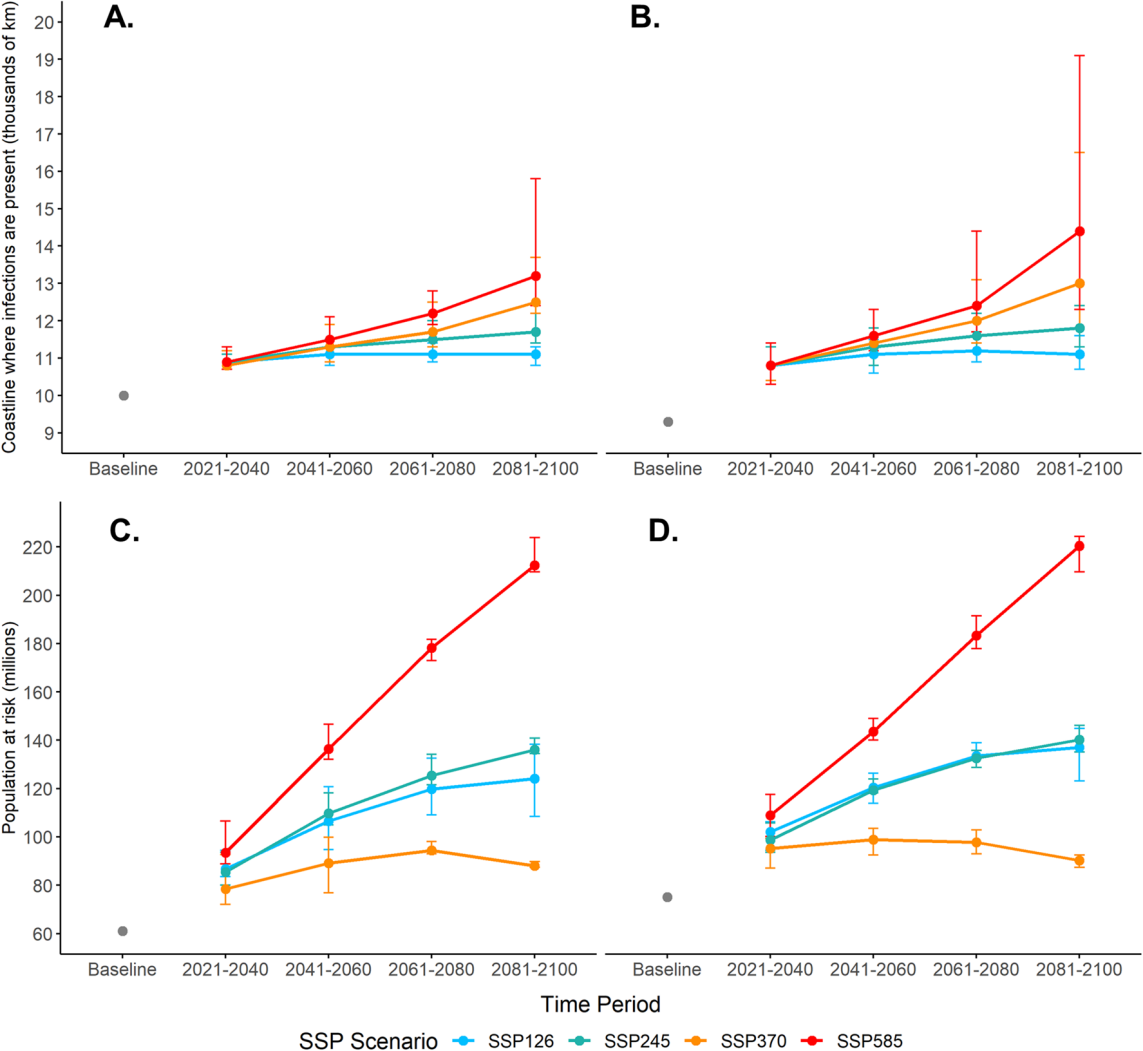
Length of the coastline where *V. vulnificus* infections are present (thousands of km) for the tmean (**A**) and tmax model (**B**). Total population at risk (millions) for the tmean (**C**) and tmax model (**D**). Future values represent an average across seven CMIP6 GCMs. Error bars represent the highest and lowest estimate from individual GCM predictions.

Figure 5 presents the length of coastline within the infection distribution and population at risk for every SSP and time period. SSPs incorporate changes in climate and changes in population. Table 1 focusses on SSP126 and SSP370 for 2041–2060, and 2081–2100 and indicates that *V. vulnificus* infections are currently present along ~ 10,000 km of the eastern USA coastline. Tmax produced a lower baseline of infection distribution estimate than tmean (9000 vs 10,000 km). In the near future (i.e., 2021–2040), the length of the coastline where infections are present increases to between 10,800 and 10,900 km under all scenarios. After this increase, the coastline lengths where infections are present diverges depending upon SSP. By 2041–2060, *V. vulnificus* infections may be present along ~ 11,100 km (SSP126) to ~ 11,500 km (SSP585) of USA coastline. However, far future predictions indicate a ~ 2100 km difference in coastline where infections are present between SSP126 and SSP585. Under SSP585, the tmax model (2081–2100) indicates 14,400 km of coastline where infections are present and stretches as far North as the St Lawrence River. However, this is an unlikely worst-case scenario that is at odds with increasing clean energy use^22^.

**Table 1:** Length of coastline where *V. vulnificus* infections are present (thousand km) population at risk (millions), percentage of population aged ≥ 60 and estimated annual number of *V. vulnificus* cases under CMIP6 Shared Socioeconomic Pathways SSP126 and SSP370.

Model	Scenario	Time period	Coastline where infections are present (thousands of km)	Total projected population at risk (millions)	Percentage of projected population at risk aged ≥ 60 years (%)	Estimated annual total number of cases
tmean	Baseline	2007–2018	10.0	61.0	16.9	61
	SSP126	2041–2060	11.1 [10.8–11.3]	106.6 [94.8–120.7]	32.5 [32.7–32.4]	145 [130–164]
		2081–2100	11.1 [10.8–11.3]	124.1 [108.4–138.3]	43.1 [43.3–42.9]	204 [178–228]
	SSP370	2041–2060	11.3 [10.9–11.9]	89.1 [76.9–99.9]	31.4 [31.5–31.3]	120 [104–135]
		2081–2100	12.5 [12.2–13.7]	88.0 [87.4–89.7]	41.1 [41.1–40.8]	141 [141–143]
tmax	Baseline	2007–2018	9.3	75.1	16.9	76
	SSP126	2041–2060	11.1 [10.6–11.5]	120.5 [113.9–126.3]	32.4 [32.5–32.4]	164 [156–172]
		2081–2100	11.1 [10.7–11.6]	137.0 [123.2–144.8]	42.9 [43.1–42.9]	224 [202–237]
	SSP370	2041–2060	11.4 [11.1–12.3]	98.9 [92.5–103.5]	31.3 [31.4–31.3]	133 [125–140]
		2081–2100	13.0 [11.8–16.5]	90.2 [87.4–92.5]	40.4 [41.1–39.6]	142 [141–144]

Values are the ensemble mean from seven global climate models and the minimum and maximum estimates are given in square brackets.

When the coastline where infections are present is combined with the SSP-specific population, the impacts diverge further between SSPs. These impacts combine climate change and population change. Under all SSPs there is a large increase in population at risk between baseline and 2021–2040 as New York and nearby states become incorporated. By 2081–2100 the population at risk varies between ~ 210 million (SSP585) and ~ 90 million (SSP370). The tailing off of population at risk for many SSPs between 2041–2060 and 2081–2100 is notable. This is due to reductions in population associated with these SSPs^24^ and for SSP370, this represents a reduction in the population at risk in spite of a larger coastline where *V. vulnificus* infections are present, due to a lower future population under SSP3 particularly in NE USA^25^.

Aged populations are more susceptible to *V. vulnificus* infections and when the projected population aged over 60 is calculated large increases in this age group are observed across all models from 17% at baseline to 31% by 2041–2060, and over 40% by 2081–2100. When the estimated number of cases is calculated based upon current age specific case rates (20-year age bands) the results indicate a case doubling by 2041–2060 under most SSPs and models. By 2081–2100 cases increase again to more than 3 times baseline under SSP126. Under SSP370 by 2081–2100 there is a smaller increase over 2041–2060 numbers due to the smaller future population under this SSP. Currently the case fatality rate for *V. vulnificus* wound infections is around 18%^12^. The projections combine climate change and population change and in Table S4 these are replicated focussing on population change only (i.e., *V. vulnificus* distribution is held constant). For SSP126 around half the change in case numbers is associated with climate change and the other half population change. For SSP370 between 67 and 75% of the change is climate related.

## Discussion

Our projections of *V. vulnificus* infection distribution and population at risk have multiple strengths. They are based upon one of the most detailed databases of *V. vulnificus* cases to date (Cholera and Other *Vibrio* Illness Surveillance [COVIS]^26^), which covers the Gulf and Atlantic USA coasts spanning 30 years (1988–2018). COVIS data trained and validated the models which were generated using 100 different samples of historical data and had strong predictive power. Each GCM produced similar *V. vulnificus* infection distributions and uncertainty is presented as the range between different GCMs.

*Vibrio vulnificus* is an increasingly recognized human pathogen with a low incidence but a high wound infection fatality rate of ~ 18%^12^. Here we show that in Eastern USA the total reported *V. vulnificus* cases have increased eightfold between 1988 and 2018, accompanied by a profound geographical expansion. In the late 1980’s infections were rare above Georgia (32°N) but by 2018 were regularly reported as far north as Philadelphia (40°N). On average the *V. vulnificus* infection distribution has been shifting northwards at ~ 48 km p.a. Our analyses confirm studies which have documented *Vibrio* infection emergence in new US locations^6^. Between 1988 and 2016 there have been over 1100 wound infections reported in the USA with 159 associated fatalities, highlighting the significant yet underappreciated impact of this pathogen.

Our projections indicate that climate change will have a major effect on *V. vulnificus* infection distribution and number in Eastern USA, likely due to warming coastal waters favouring presence of bacteria and elevated temperatures leading to more coastal recreation. By 2041–2060 the coastline where *V. vulnificus* infections are present increases by over 1000 km under both SSP126 and SSP370 and both tmean and tmax models. This shift increases the population at risk into the densely populated coastal regions of New Jersey and New York. Alongside population growth and an increasingly elderly population, this translates into a doubling of cases by 2041–2060.

By 2081–2100 the patterns increasingly diverge between SSPs. Under SSP126 the coastline where *V. vulnificus* infections are present remains relatively static but increases in the elderly population under this ‘sustainability’ scenario led to further increases in the projected population at risk and cases. Conversely, under SSP370 the coastline where *V. vulnificus* infections are present increases by another 1000 km into southern Maine, to encompass every state along the Eastern USA coastline. However, this shift occurs into less densely populated areas, and within SSP370 there are population reductions under this ‘regional rivalry’ scenario. For context, the population within the current *V. vulnificus* disease distribution (see Fig. 2), is projected to decrease by 9.3 million over the 21st century under the SSP3 pathways (Table S4). The overall impacts under SSP370 are a small increase in the projected number of cases in comparison to 2041–2060. Under all SSPs and time periods, tmax gave a marginally greater increase in coastline where *V. vulnificus* infections are predicted compared to tmean.

The 4° shift in the northern coastline extent where *V. vulnificus* infections are present under SSP370 projected by 2080 (~ 0.06° p.a.) is slower than the rate observed between 1988 and 2018 (0.43° p.a.). Potential reasons include steeper latitude-temperature gradients at higher latitudes or few reported cases early in the observational period (1988–2018) in mid-latitudes. This is plausible due to the rare nature of a *V. vulnificus* illness intersecting an area of lower population (i.e., North and South Carolina, Fig. 1).

Our work uses an ENM to evaluate *V. vulnificus* infection spread. The best model is based upon air temperature, a surrogate for both sea surface temperature (the two are strongly correlated^27,28^) and coastal recreation^29^. This approach contrasts to previous studies^9^ which focus on the ecological suitability for the *V. vulnificus* bacteria, which even if present, may not lead to human disease^30^. Therefore, within our current and future *V. vulnificus* disease distribution there will be coastal areas where conditions are unsuitable for *V. vulnificus* bacteria. However, it is worth noting that despite this limitation *V. vulnificus* infections currently occur along the USA coastline from Southern Texas to Maine (Fig. 1). The current and future oceanographic data resolution (25 × 25 km) is a particular challenge for coastal studies as the underlying observations are often poorly representative of near coastal conditions where *V. vulnificus* thrive and ultimately come into contact with the human population.

This work has uncertainties. Exposure case location was assumed as the nearest coastline to an individual’s home/travel county, but case location was only used to define the current *V. vulnificus* distribution. Population at risk was based upon residents within 200 km of the coast (~ 2-h travel time), but different distance buffers could have been used. Projected cases were based upon current age-specific incidence rates, but these may change. The analysis used future downscaled climate data, bias corrected with current observational data, but future weather patterns may be different. We were unable to model changes to the *V. vulnificus* southern extent, but this bacterium has been isolated from oysters as far south as Tabasco (18.5°N)^31^.

The northward *V. vulnificus* infection expansion stresses the need for increased individual and public health *V. vulnificus* awareness in these areas. This is crucial as prompt action when symptoms occur is necessary to prevent major health outcomes^32^. Individuals and health authorities could be warned in real time about particularly risky environmental conditions through marine^33^ or *Vibrio*-specific early warning systems^34^. Active control measures could include greater awareness programmes for at risk groups (e.g. the elderly, individuals with underlying conditions), and coastal signage during high-risk periods.

## Methods

### *Vibrio vulnificus* data

Since 1988 CDC has maintained the Cholera and Other *Vibrio* Illness Surveillance database (COVIS) for the reporting of human cases of vibriosis and cholera. Laboratory-confirmed cases of *V. vulnificus*, where the transmission route was confirmed as non-foodborne, foreign travel was not reported, and the patient did not live in the Pacific Region of the US (or travel outside this Region) were extracted (1375 cases) for the years 1988–2018. For a few cases (69) a symptom date was not present, but in all but 3 cases one was generated by applying the modal lag between cases where a symptom date and specimen date were present.

An accurate location of exposure is important, and for most cases this was based on the city/county where the individual lived or travelled to in the days preceding symptoms. Cases were excluded (128) where the home/ travel location was coarser than city/county. A further 75 cases were excluded as the home/travel location was further than 200 km from the coast (> 2-h drive), introducing uncertainty into location of exposure which is predominantly coastal. The analysis proceeded with 1169 cases (85% of total) which were matched to their nearest coastline. Equivalent *V. vulnificus* data from Canada were unavailable, and an absence of case reports in the literature strongly suggests negligeable incidence.

### Baseline oceanographic, climate and climate change projection data

*Vibrio vulnificus* is known to be affected by both sea surface temperature (SST) and seawater salinity. Gridded historical and future data sets of SST and salinity were downloaded for the Alfred Wegener Institute Climate Model (AWI-CM-1-1-MR) at a spatial resolution of 25 km^35^. Historical data were downloaded between 2007 and 2014 (data were not available beyond 2014). Future data were downloaded for the years 2018–2100 under SSP126, SSP245, SSP370 and SSP585. For baseline and all future time periods (2021–2040, 2041–2060, 2061–2080 and 2081–2100) the mean monthly temperature for each calendar month during the period of interest was calculated. The mean and maximum salinity and mean and maximum SST were then calculated from these 12 values.

Meteorological conditions such as air temperature and precipitation not only influence SST and salinity (SST and air temperature are highly correlated^27,28^) but can also affect human behaviour and hence exposure to *V. vulnificus*. They are also known to influence coastal recreational behaviour^29^. Crucially they are available at finer grid resolutions. For baseline conditions, gridded historical monthly maximum air temperature (°C) and monthly total precipitation (mm) were obtained from the WorldClim database for the years 2007 to 2018. The air temperature data^36^ which has been bias corrected using WorldClim 2.1^37^ to a spatial resolution of 2.5 arcminutes (~ 4.6 km), represents monthly means of the daily maximum air temperatures. Future maximum air temperature (°C) and total precipitation (mm) were obtained from the WorldClim ‘Future Climate’ dataset which had been downscaled and bias-corrected with the same WorldClim 2.1 baseline^37^. These data were available for four future 20-year time periods under four SSPs as gridded monthly averages across each 20-year period for January to December, respectively. Individual data were obtained for seven GCMs (BCC-CSM2-MR, CNRM-CM6-1, CNRM-ESM2-1, CanESM5, IPSL-CM6A-LR, MIROC-ES2L, MIROC6) downscaled at a spatial resolution of 2.5 arcminutes (~ 4.6 km) (see Table S1 for GCM references).

To ensure the compatibility of historical and future temperature data, monthly averages of maximum air temperature were calculated for each calendar month across the 11-year historical period of 2007 to 2018. Therefore, each grid cell contained 12 values (one average value per calendar month) for the historical period and 12 for a given future time period.

From the air temperature data tmean was calculated as the average of 12-monthly values for each time period. The maximum of these values was also calculated. Using the monthly total precipitation, mean precipitation, and maximum precipitation variables were also calculated in the same way.

### Historic and future population scenario data

Baseline population at risk and age distribution was calculated using 2010 subsets of Gridded Population of the World, Version 4: Population Count and Gridded Population of the World, Version 4: Basic Demographic Characteristics, respectively^38,39^, at a resolution of 2.5 arcminutes (~ 4.6 km). These data were subdivided into 20-year age categories (0–19, 20–39, 40–59, 60 and older).

SSP-specific future population data were obtained at the same spatial resolution as annual projections^24^. These data were subdivided into age categories using SSP-specific future U.S. County-Level Population Projections^40^.

### Changing distribution

To assess whether the geographical distribution of cases has shifted over time, the mean latitude, 5th percentile (southern extent) and 95th percentile of latitude (northern extent) was calculated for the dataset in annual time steps. These data were plotted and trends assessed.

### Model specification and creation

The current spatial distribution of *V. vulnificus* cases (presence:absence) was calculated using cases from 2007 onwards (n = 709) to ensure a contemporary distribution and because *V. vulnificus* became notifiable in 2007 (therefore there was potential for earlier cases to be unreported). The northern extent of the distribution was set as the 95th percentile latitude of cases (39.93°N adjacent to Philadelphia). The distribution was assumed to extend southwards along the US coastline to the Mexican border (25.95°N; cases were reported along the entire coastline to this border; Fig. 1), as results indicated no change in the southern extent and *V. vulnificus* has been isolated in shellfish throughout the Gulf of Mexico. The coastline absent of *V. vulnificus* infections was defined as northwards from 39.93°N to the northernmost point of Newfoundland and Labrador located (60.35°N). Grid cells directly intersecting the coastline were used to define presence:absence locations (oceanographic data, SST and salinity, 318 presence cells, 596 absence cells; climate data, air temperature and precipitation, 2990 presence cells, 5252 absence cells).

The creation of each model was based upon cells along the coast that were labelled as present/absent for *V. vulnificus*. The oceanographic (SST and salinity) and climate (air temperature and precipitation) data were assigned to each cell. Models were generated using oceanographic and climate data. For each model, the proportion of presence:absence points was kept constant and 10% of the data were set aside for model validation whilst the remaining 90% were carried forward for model creation and testing. From this remaining 90% of the data, 100 random samples were obtained which contained all the presence points and an identical number of randomly selected absence points to ensure that the models were not biased towards predicting either presence or absence. Each of the 100 random samples of data were split 70:30 into training and testing subsets whilst maintaining a 50:50 ratio of presence: absence points in both training and testing subsets. Binomial logistic regression models of *V. vulnificus* presence-absence were fitted using tenfold cross validation on each of the 100 training subsets using a Generalised Linear Model (GLM) method within the package ‘caret’^41^ in R version 4.0.2^42^. Model predictive power was measured using the mean AUC calculated for each of the 100 model replicates on the corresponding testing subsets using R package ‘pROC’^43^. Multiple variations of oceanographic and climate variables were fitted as univariate models, and each was tested to check that the assumption of linearity between the predictor variable and logit of the outcome had been met. All coefficients and metrics of model performance were averaged over the 100 replicates. Each model produced this way (i.e., as an average of the 100 replicates) was used to predict on the corresponding 10% validation dataset which had been held out of the model creation process for each variable tested. A further AUC was generated to check the ability of the model to predict on unseen data, this is referred to as the ‘Validation AUC’.

### Distribution maps

After the final models were selected, predictors from the historical data set and from every future SSP / time period combination were passed through the model to produce estimates of current and future *V. vulnificus* distribution. For the future time period the output from all 7 GCMs were averaged to generate a multi-model mean prediction of the distribution. The model outputs were the probability of *V. vulnificus* presence and these probabilities were converted into a binary map using the ‘PresenceAbsence’ package^44^ in R version 4.0.2^42^. This package translates probability of occurrence into a binary (presence:absence) parameter based on a given ‘Required Sensitivity’. A required sensitivity of 0.85 (ReqSens85) was chosen to ensure that that no more than 15% of risk locations were missed whilst maintaining the highest degree of specificity possible. The ReqSens85 threshold meant that probabilities greater than or equal to 0.999 were classified as presence and probabilities below 0.999 as absences. A lower Required Sensitivity threshold was not applied as *V. vulnificus* is a rare infection and it is important not to misidentify true locations as absences^45^. Modifying this parameter made negligeable difference to the distribution due to the strong predictive power of the chosen models (tmean AUC 0.999, tmax AUC 0.998).

### Coastlines, population at risk and projected cases

The sum of the coastline length within climate cells where presence was calculated for each SSP scenario and future time period. The population at risk of *V. vulnificus* infection was determined as the sum of the population that reside within a 200 km buffer surrounding these coastal cells and clipped to the maximum latitude of predicted risk (to be consistent with the method used to assign cases to coastal locations). To estimate likely cases, the current case rates by 20-year age bands (0–19, 20–39, 40–59, 60 and older) were calculated using age data within COVIS. These rates were multiplied by the age profile in the risk zone (200 km buffer surrounding coastline where *V. vulnificus* infections are present) to estimate likely baseline and future cases.

## Supplementary Information

Supplementary Information.

## Data Availability

The Cholera and Other *Vibrio* Illness Surveillance database (COVIS) may be obtained through enquiry to the US Center for Disease Control, Atlanta, Georgia. The oceanographic and climate data may be freely obtained from the CMIP6 CEDA ESGF search portal for the Alfred Wegener Institute and the WorldClim website. Population data may be obtained from the Gridded Population of the World, Version 4 (GPWv4). SSP specific future population data were obtained from the ISIMIP ESGF server. SSP specific future age distributions were obtained from the U.S. County-Level Population Projections^40^.
