# Supplementary material for: Climate warming and increasing Vibrio vulnificus infections in North America
Source: Sci Rep. 2023 Mar 23;13:3893. doi: 10.1038/s41598-023-28247-2 (PMC10036314; doi:10.1038/s41598-023-28247-2)
Supplement: Supplementary file 1 — Supplementary Information. [file 41598_2023_28247_MOESM1_ESM.pdf]

Supplementary Material

Table S1. Global Climate Models (GCMs) used in this study.

Model Name	Institution(s)	Main Reference(s)
AWI-CM-1-1-MR	Alfred Wegener Institute	(Semmler *et al.*, 2020)[1]
BCC-CSM2-MR	Beijing Climate Center	(Xin *et al.*, 2018)[2]
CNRM-CM6-1	Centre National de Recherches Meteorologiques and Centre Europeen de Recherche et Formation Avancees en Calcul Scientifique	(Voldoire, 2018; Voldoire *et al.*, 2019)[3,4]
CNRM-ESM2-1	Centre National de Recherches Meteorologiques and Centre Europeen de Recherche et Formation Avancees en Calcul Scientifique	(Séférian *et al.*, 2019)[5]
CanESM5	Canadian Center for Climate Modelling and Analysis	(Swart *et al.*, 2019)[6]
IPSL-CM6A-LR	Institut Pierre-Simon Laplace Climate Modelling Centre	(Boucher *et al.*, 2020)[7]
MIROC-ES2L	Japan Agency for Marine-Earth Science and Technology; Atmosphere and Ocean Research Institute, The University of Tokyo; National Institute for Environmental Studies; RIKEN Center for Computational Science	(Hajima *et al.*, 2020)[8]
MIROC6	Japan Agency for Marine-Earth Science and Technology; Atmosphere and Ocean Research Institute, The University of Tokyo; National Institute for Environmental Studies; RIKEN Center for Computational Science	(Tatebe *et al.*, 2019)[9]

Table S2. Length of coastline where *V. vulnificus* infections are present (thousand km) population at risk (millions), percentage of population aged ≥ 60 and estimated annual number of *V. vulnificus* cases under CMIP6 Shared Socioeconomic Pathways SSP126, SSP245, SSP370 and SSP585. Values are for the tmean model and are the ensemble mean from seven global climate models and the minimum and maximum estimates are given in square brackets.

Scenario	Time Period	Coastline where infections are present (thousands of km)	Total projected population at risk (millions)	Percentage of projected population at risk aged ≥60 years (%)	Estimated annual total number of cases
**Baseline**	**2007 - 2018**	10.0	61.0	16.9	61
**SSP126**	**2021 - 2040**	10.9 [10.7 - 11.1]	86.7 [83.6 - 94.4]	27.2 [27.2-27.2]	106 [102-115]
	**2041 - 2060**	11.1 [10.8-11.3]	106.6 [94.8-120.7]	32.5 [32.7-32.4]	145 [130-164]
	**2061 - 2080**	11.1 [10.9 - 11.4]	119.8 [109.1 - 132.6]	43.0 [43.2-42.9]	196 [179-216]
	**2081 - 2100**	11.1 [10.8-11.3]	124.1 [108.4-138.3]	43.1 [43.3-42.9]	204 [178-228]
**SSP245**	**2021 - 2040**	10.9 [10.7 - 11.1]	85.4 [80.1 - 93.6]	26.3 [26.3-26.2]	102 [95-112]
	**2041 - 2060**	11.3 [11.1 - 11.5]	109.7 [105.0 - 118.2]	30.1 [30.1-30.0]	142 [136-153]
	**2061 - 2080**	11.5 [11.3 - 12.0]	125.4 [121.5 - 134.1]	38.8 [38.8-38.7]	192 [186-204]
	**2081 - 2100**	11.7 [11.4 - 12.2]	136.0 [134.5 - 140.9]	38.7 [38.8-38.7]	208 [205-215]
**SSP370**	**2021 - 2040**	10.8 [10.7 - 11.2]	78.4 [72.0 - 92.6]	27.0 [27.0-27.0]	95 [88-113]
	**2041 - 2060**	11.3 [10.9-11.9]	89.1 [76.9–99.9]	31.4 [31.5-31.3]	120 [104-135]
	**2061 - 2080**	11.7 [11.3 - 12.5]	94.4 [92.9 - 98.0]	41.2 [41.2-41.1]	152 [149-158]
	**2081 - 2100**	12.5 [12.2-13.7]	88.0 [87.4-89.7]	41.1 [41.1-40.8]	141 [141-143]
**SSP585**	**2021 - 2040**	10.9 [10.7- 11.3]	93.5 [88.8 - 106.5]	25.7 [25.7-25.8]	110 [103-124]
	**2041 - 2060**	11.5 [11.3 - 12.1]	136.4 [132.1 - 146.6]	28.9 [28.9-28.8]	170 [165-182]
	**2061 - 2080**	12.2 [11.9 - 12.8]	178.2 [172.9 - 181.7 [2]]	35.2 [35.2-35.2]	251 [244-257]
	**2081 - 2100**	13.2 [12.4 - 15.8]	212.4 [209.6 - 223.7]	34.9 [35.2-33.4]	298 [296-300]

Table S3. As Table S1 but for the tmax model.

Scenario	Time Period	coastline infections are present (thousands of km)	Total projected population at risk (millions)	Percentage of projected population at risk aged ≥60 years (%)	Estimated annual total number of cases
**Baseline**	**2007 - 2018**	9.3	75.1	16.9	76
**SSP126**	**2021 - 2040**	10.8 [10.4 - 11.3]	102.0 [94.4 - 106.3]	27.3 [27.2-27.4]	124 [115-131]
	**2041 - 2060**	11.1 [10.6-11.5]	120.5 [113.9-126.3]	32.4 [32.5-32.4]	164 [156-172]
	**2061 - 2080**	11.2 [10.9 - 11.6]	133.5 [132.6 - 138.9]	42.9 [42.9-42.9]	218 [216-228]
	**2081 - 2100**	11.1 [10.7-11.6]	137.0 [123.2-144.8]	42.9 [43.1-42.9]	224 [202-237]
**SSP245**	**2021 - 2040**	10.8 [10.4 - 11.3]	98.7 [93.6 - 105.8]	26.3 [26.2-26.4]	117 [112-126]
	**2041 - 2060**	11.3 [10.8 - 11.8]	119.3 [118.2 - 123.9]	30.0 [30.0-30.0]	154 [153-159]
	**2061 - 2080**	11.6 [11.2 - 12.2]	132.5 [128.7 - 135.8]	38.7 [38.8-38.7]	203 [197-208]
	**2081 - 2100**	11.8 [11.3 - 12.4]	140.3 [135.1 - 146.1]	38.6 [38.7-38.0]	213 [207-218]
**SSP370**	**2021 - 2040**	10.8 [10.4 - 11.4]	95.2 [87.1 - 99.4]	27.0 [26.9-27.0]	116 [105-121]
	**2041 - 2060**	11.4 [11.1-12.3]	98.9 [92.5-103.5]	31.3 [31.4-31.3]	133 [125-140]
	**2061 - 2080**	12.0 [11.4 - 13.1]	97.7 [93.0 - 103.4]	40.9 [41.2-39.6]	156 [149-160]
	**2081 - 2100**	13.0 [11.8-16.5]	90.2 [87.4-92.5]	40.4 [41.1-39.6]	142 [141-144]
**SSP585**	**2021 - 2040**	10.8 [10.3 - 11.4]	108.9 [100.2 - 117.5]	25.8 [25.7-25.9]	128 [116-138]
	**2041 - 2060**	11.6 [11.2 - 12.3]	143.5 [140.0 - 149.0]	28.8 [28.8-28.9]	180 [174-187]
	**2061 - 2080**	12.4 [11.7 - 14.4	183.4 [177.9 - 191.5]	34.7 [35.2-33.5]	256 [251-258]
	**2081 - 2100**	14.4 [12.3 - 19.1]	220.4 [209.6 - 224.2]	33.8 [35.2-33.3]	299 [296-300]

Table S4. Length of coastline at risk (thousands km) population (millions) within 200 km of predicted *V. vulnificus* risk, percentage of population aged ≥ 60 and estimated annual number of *V. vulnificus* cases under CMIP6 Shared Socioeconomic Pathways SSP126 and SSP370 assuming no shift in the distribution of *V. vulnificus* (95th percentile latitude of cases: ~40°N).

Model	Scenario	Time Period	Length of coastline at risk (in thousands of km)	Total projected population at risk (in millions)	Percentage of projected population at risk aged ≥60 years (%)	Estimated annual total number of cases
No Change in the distribution of *V. Vulnificus*	**Baseline**	**2007 – 2018**	10.8	59.3	19.0	59
	**SSP126**	**2041-2060**	10.8	73.3	32.9	101
		**2081-2100**	10.8	83.3	43.6	138
	**SSP370**	**2041-2060**	10.8	59.3	31.7	80
		**2081-2100**	10.8	50.0	41.7	81

Figure S1. Boxplot of mean monthly Salinity concentration (PSU) for every 25km x 25km coastal grid cell in the study area 2007-2014 subdivided by state.

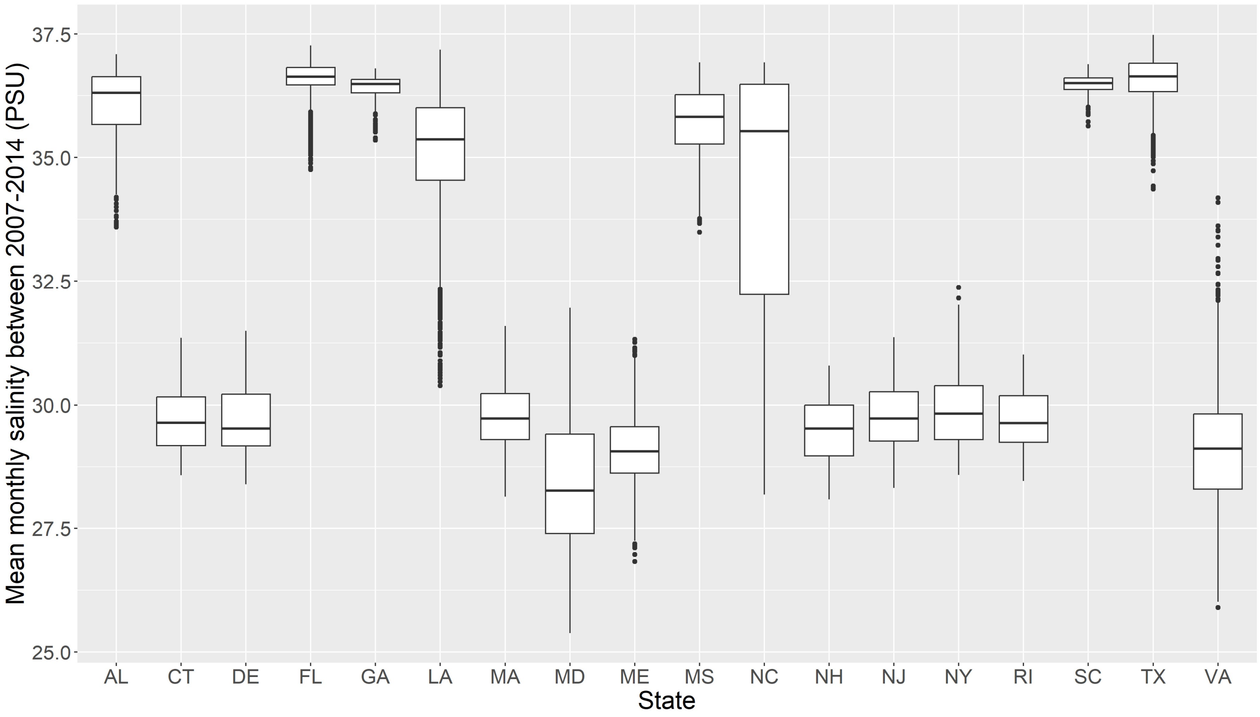

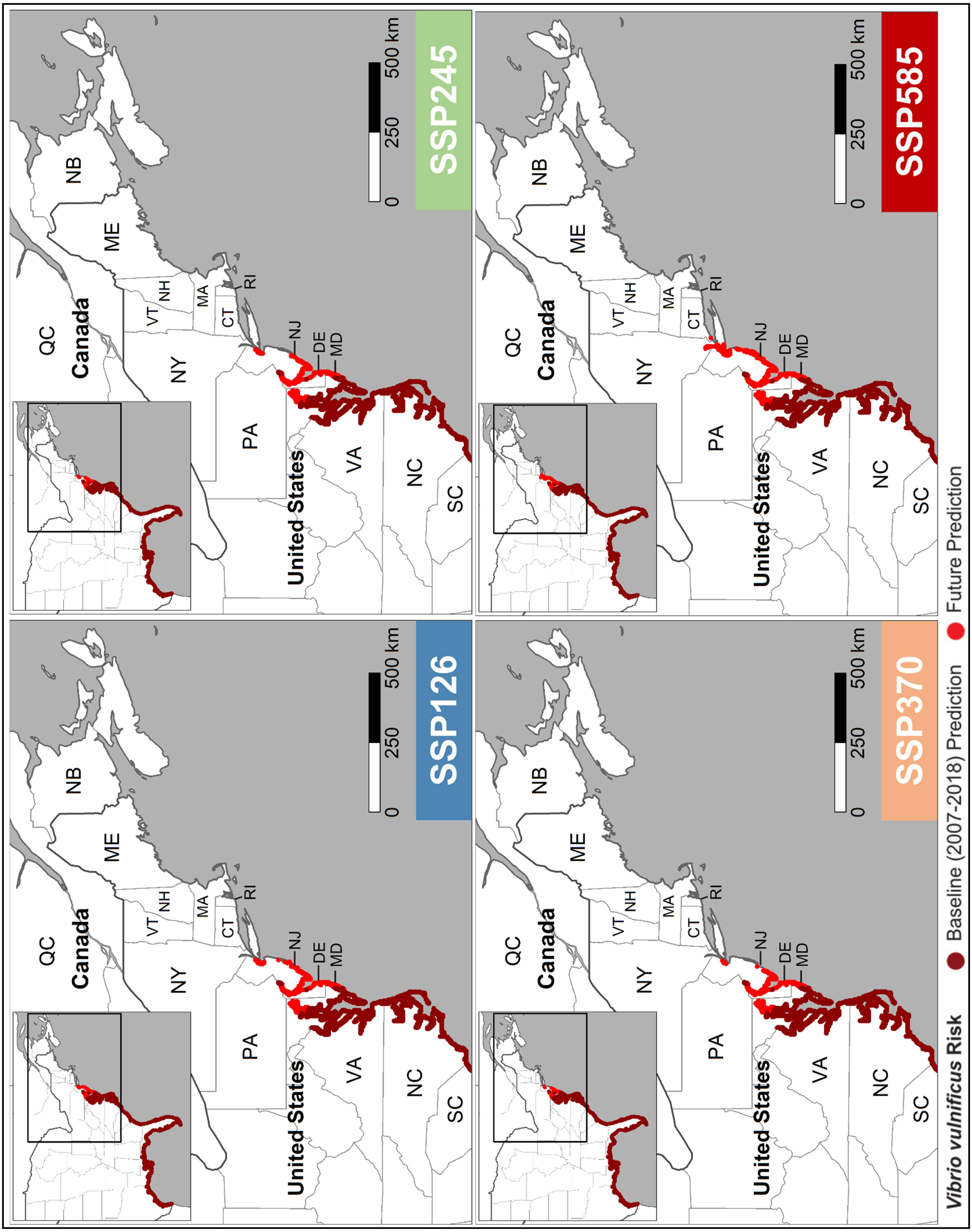

Figure S2. Tmean model prediction of *Vibrio vulnificus* human wound infection risk averaged across seven CMIP6 global climate models between 2021 – 2040 under CMIP6 Shared Socioeconomic Pathway (SSPs) SSP126, SSP245, SSP370 and SSP585. Maps generated in R version 4.0.2.

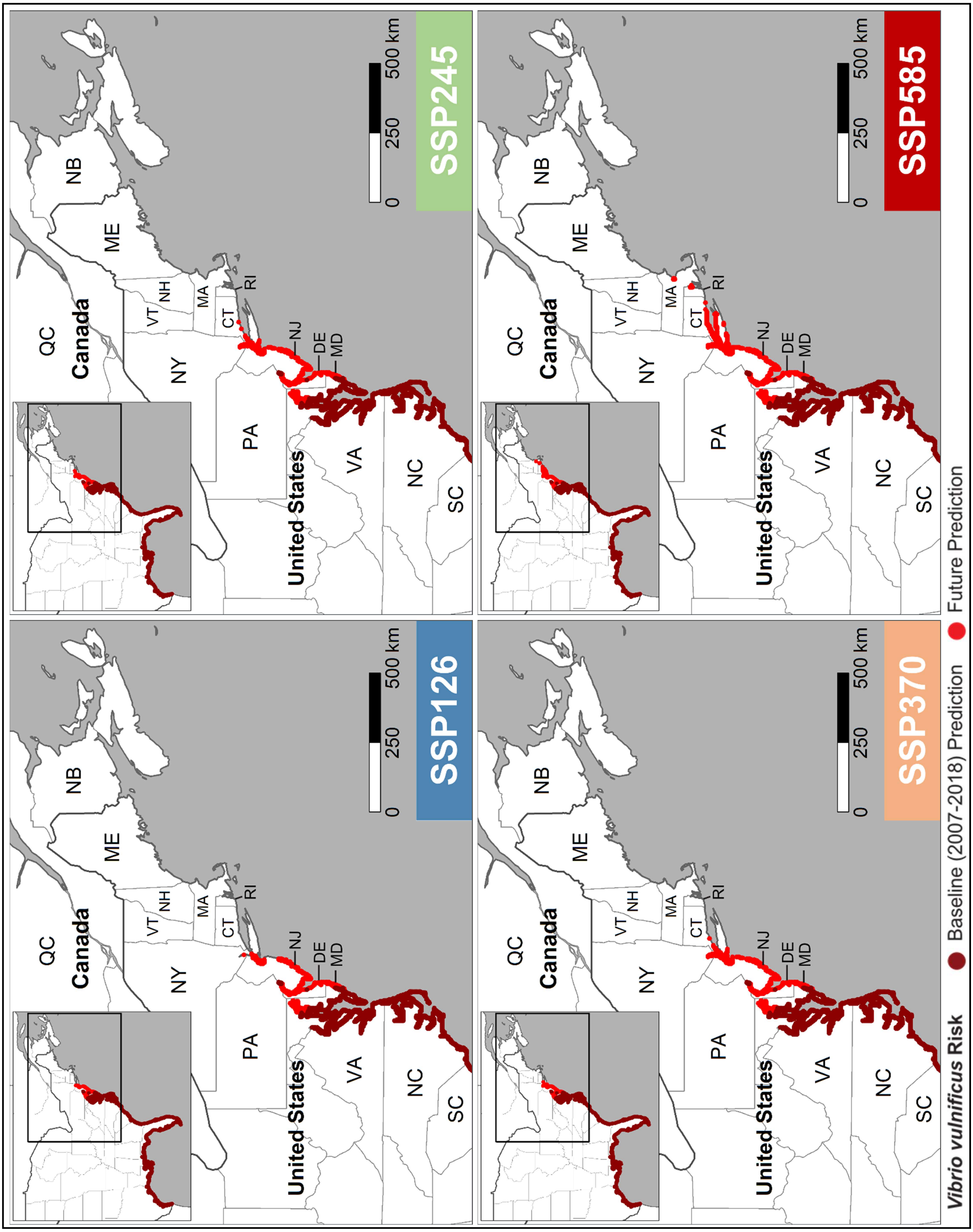

Figure S3. As Figure S2 but for 2041 – 2060.

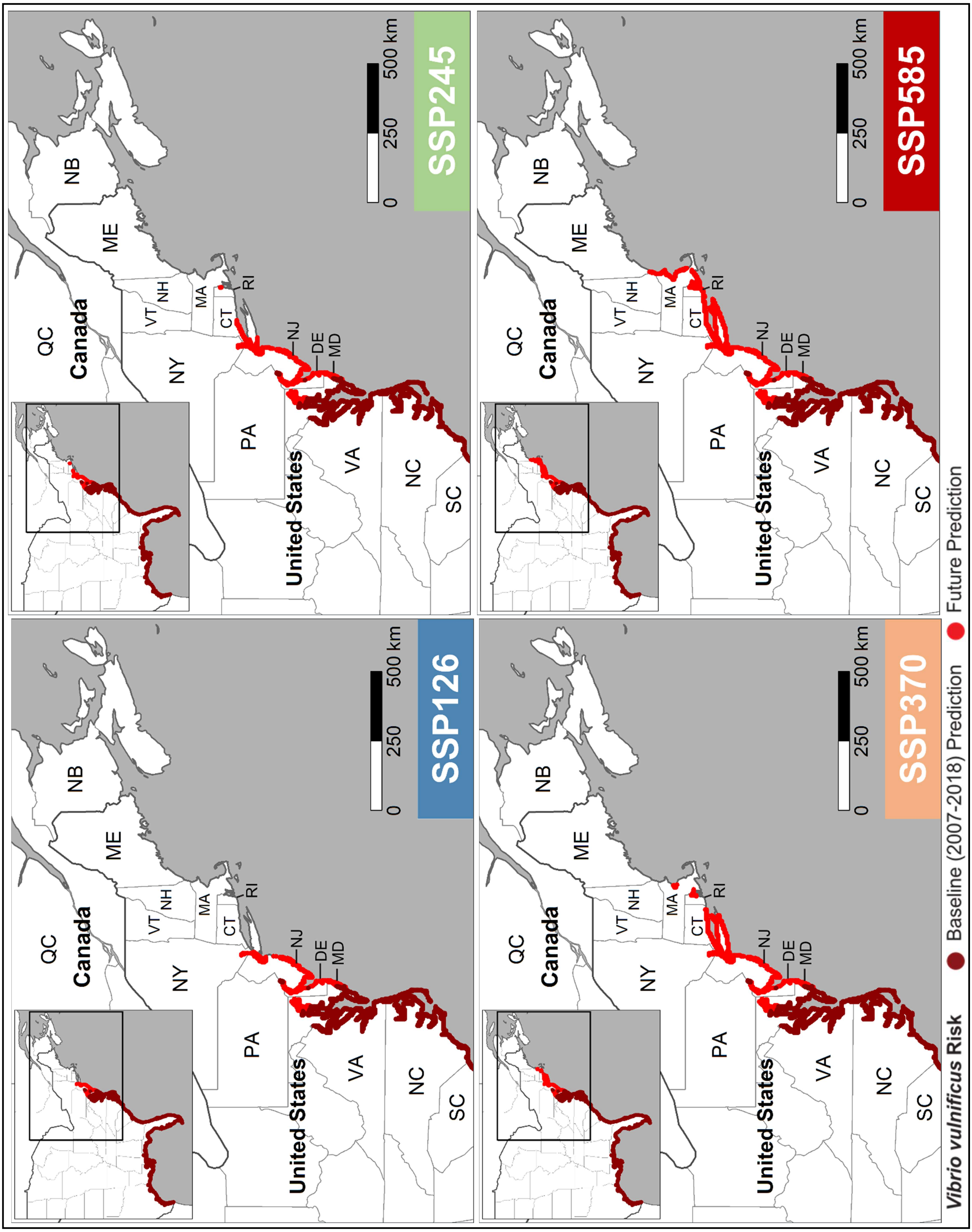

Figure S4. As Figure S2 but for 2061 – 2080.

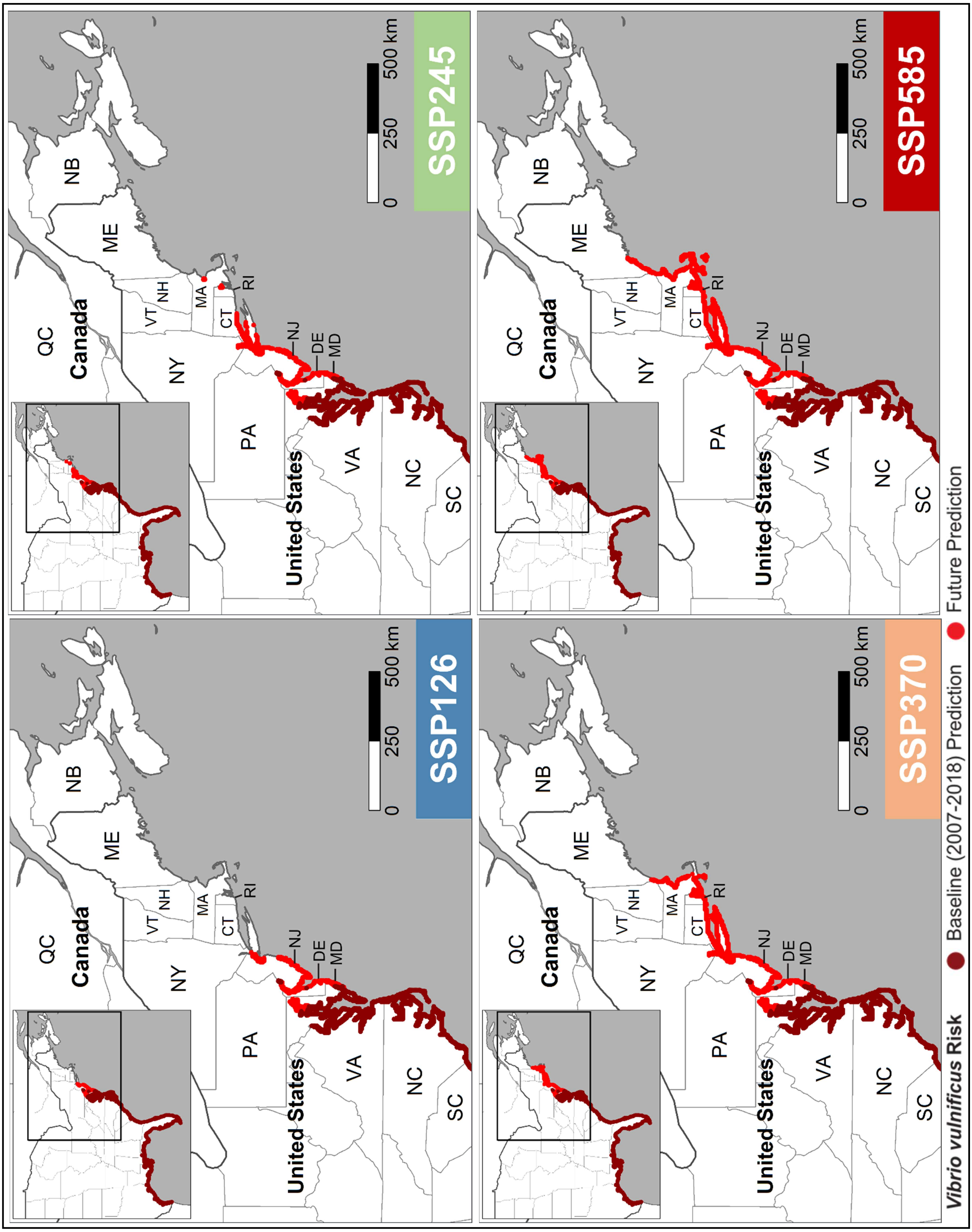

Figure S5. As Figure S2 but for 2081 – 2100.

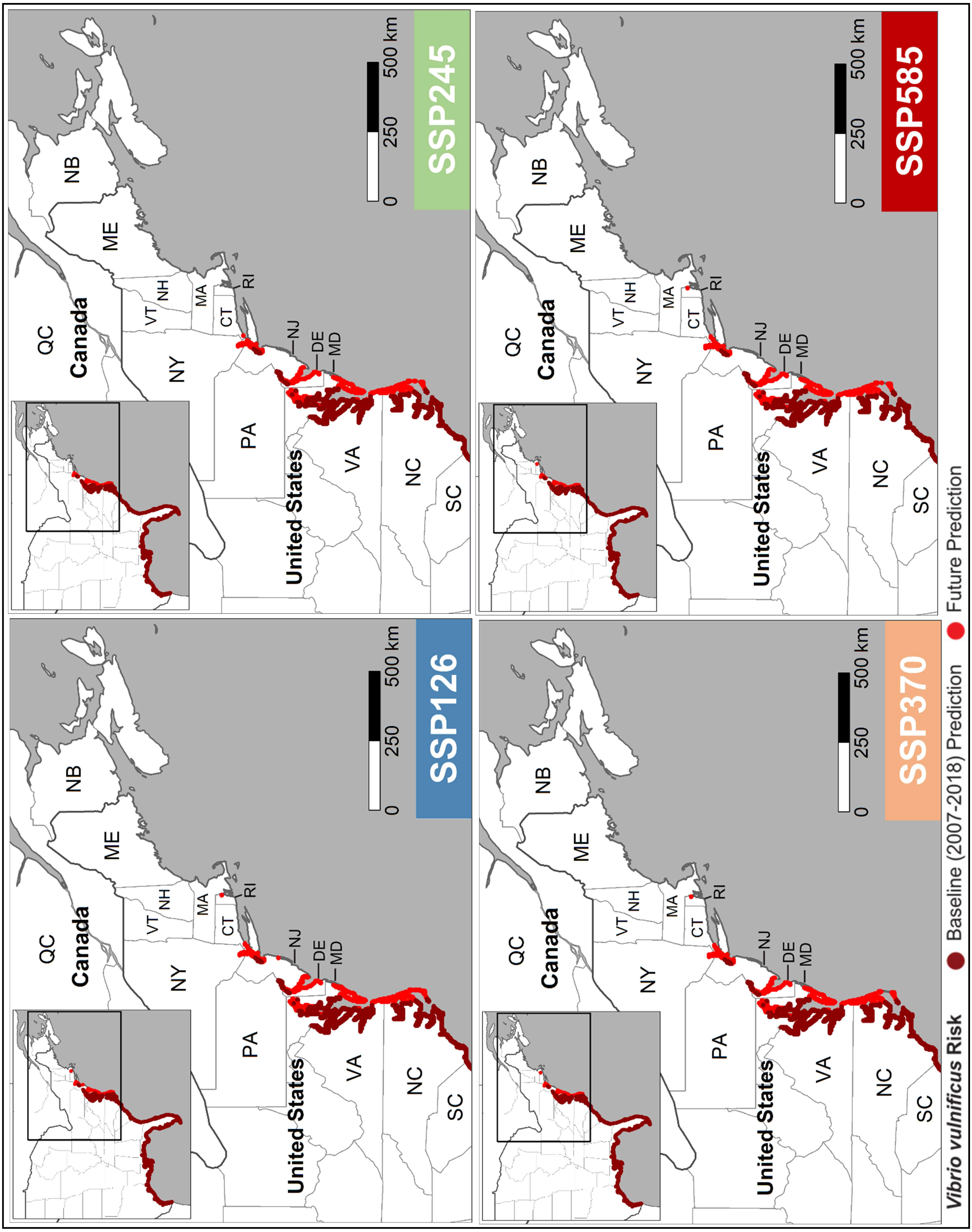

Figure S6. Tmax model prediction of *V. vulnificus* human wound infection risk averaged across seven CMIP6 global climate models between 2021 – 2040 under CMIP6 Shared Socioeconomic Pathway (SSPs) SSP126, SSP245, SSP370 and SSP585. Maps generated in R version 4.0.2.

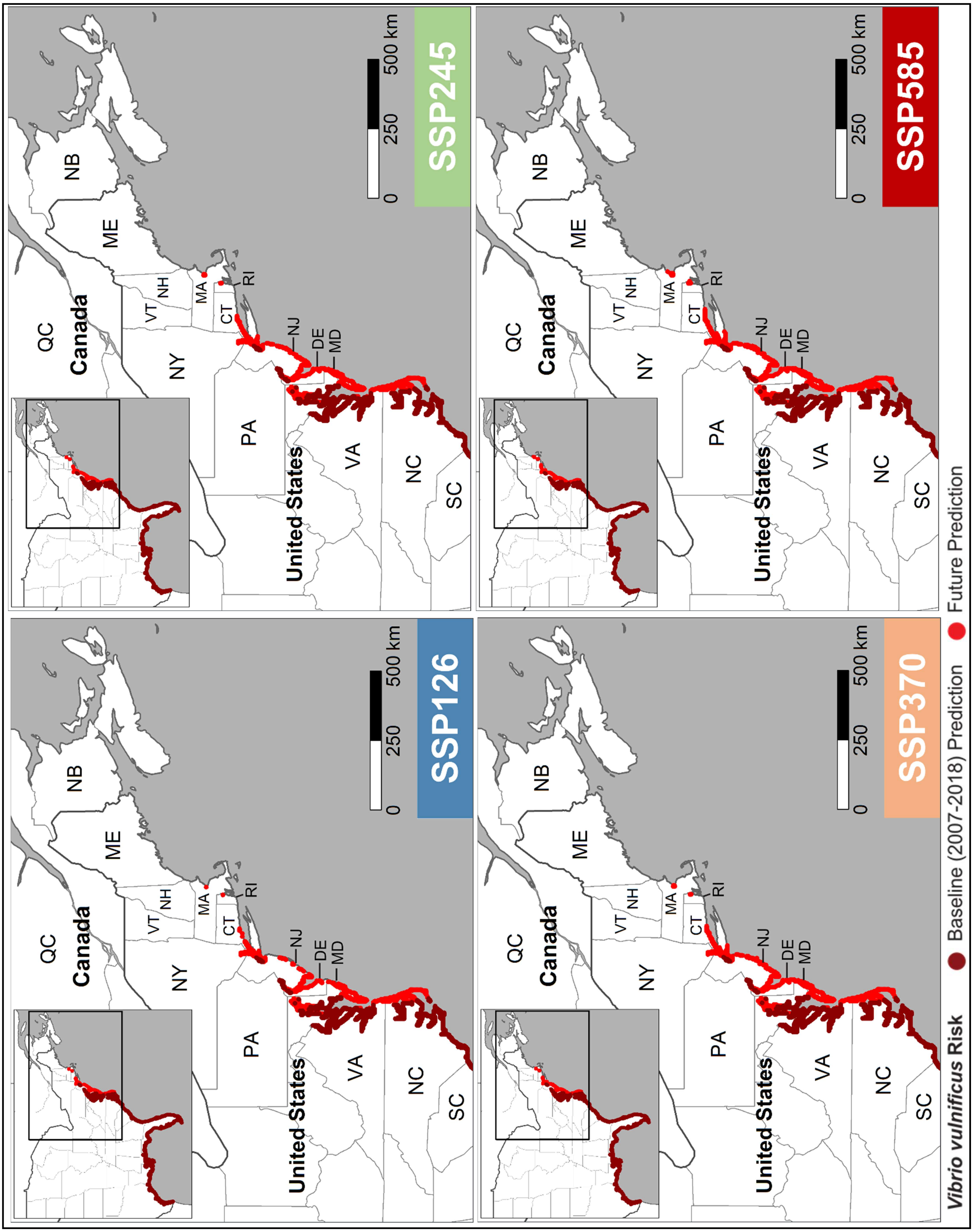

Figure S7. As Figure S6 but for 2041 – 2060.

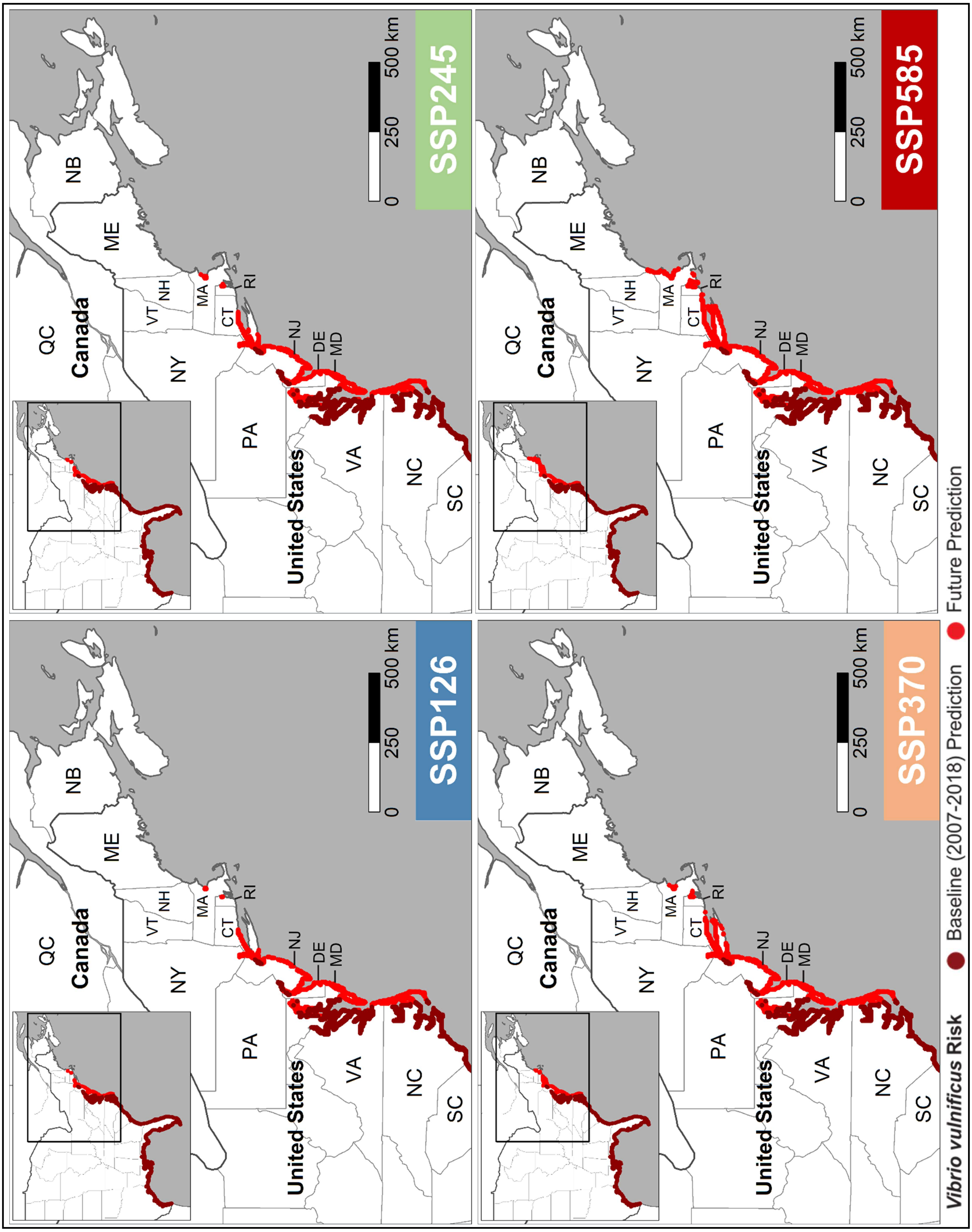

Figure S8. As Figure S6 but for 2061 – 2080.

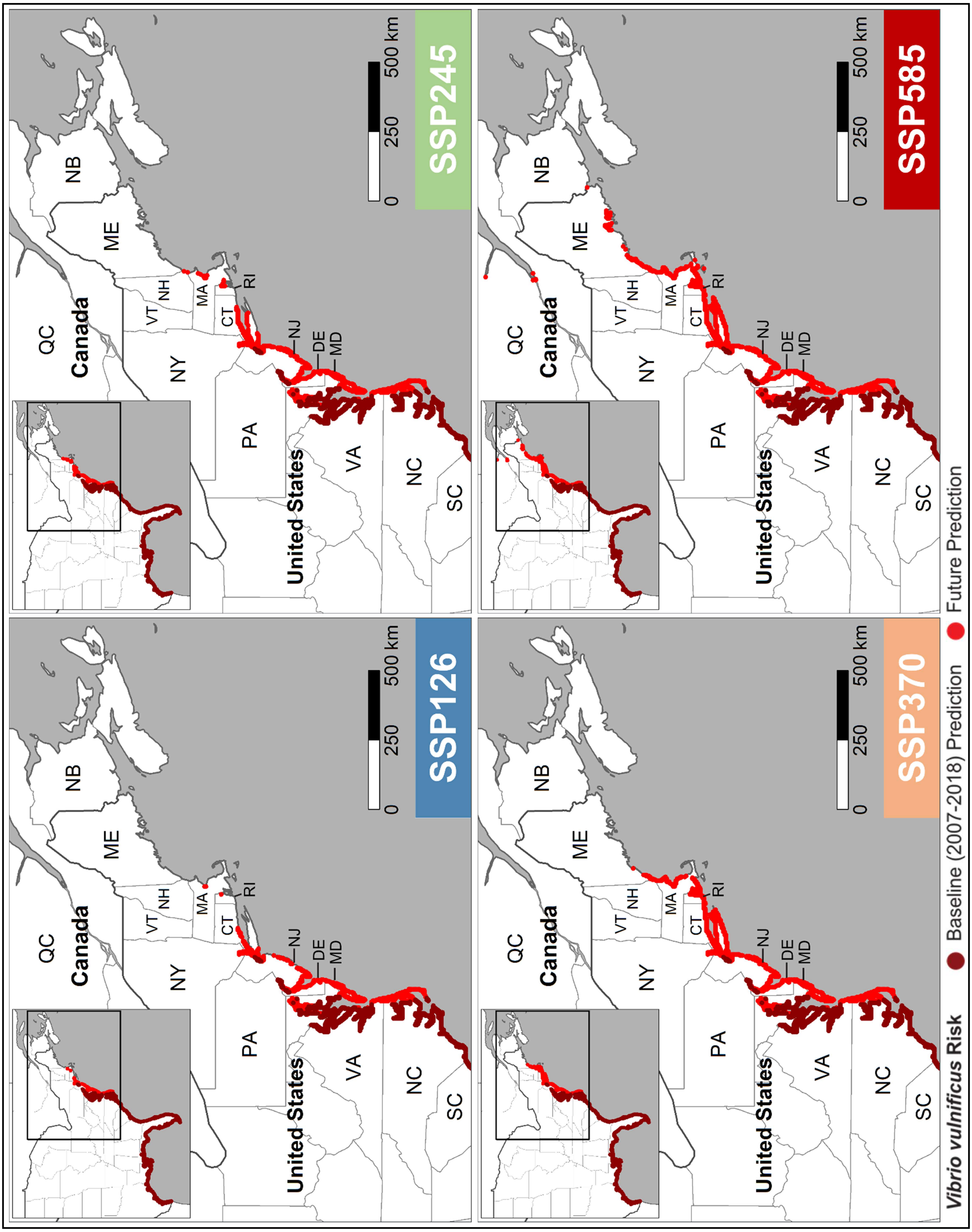

Figure S9. As Figure S6 but for 2081 – 2100.

1 Semmler, T. *et al.* Simulations for CMIP6 With the AWI Climate Model AWI-CM-1-1. *Journal of Advances in Modeling Earth Systems* **12**, doi:10.1029/2019MS002009 (2020).

2 Xin, X. *et al.* BCC BCC-CSM2MR model output prepared for CMIP6 ScenarioMIP ssp126., (2018).

3 Voldoire, A. CNRM-CERFACS CNRM-CM6-1 model output prepared for CMIP6 CMIP. (Earth System Grid Federation, 2018).

4 Voldoire, A. *et al.* Evaluation of CMIP6 DECK Experiments With CNRM-CM6-1. *Journal of Advances in Modeling Earth Systems* **11**, 2177-2213, doi:10.1029/2019MS001683 (2019).

5 Séférian, R. *et al.* Evaluation of CNRM Earth System Model, CNRM-ESM2-1: Role of Earth System Processes in Present-Day and Future Climate. *Journal of Advances in Modeling Earth Systems* **11**, 4182-4227, doi:10.1029/2019MS001791 (2019).

6 Swart, N. C. *et al.* The Canadian Earth System Model version 5 (CanESM5.0.3). *Geoscientific Model Development* **12**, 4823-4873, doi:10.5194/gmd-12-4823-2019 (2019).

7 Boucher, O. *et al.* Presentation and Evaluation of the IPSL-CM6A-LR Climate Model. *Journal of Advances in Modeling Earth Systems* **12**, doi:10.1029/2019MS002010 (2020).

8 Hajima, T. *et al.* Development of the MIROC-ES2L Earth system model and the evaluation of biogeochemical processes and feedbacks. *Geoscientific Model Development* **13**, 2197-2244, doi:10.5194/gmd-13-2197-2020 (2020).

9 Tatebe, H. *et al.* Description and basic evaluation of simulated mean state, internal variability, and climate sensitivity in MIROC6. *Geoscientific Model Development* **12**, 2727-2765, doi:10.5194/gmd-12-2727-2019 (2019).
